# Pharmacological investigation on the anti-oxidant and anti-inflammatory activity of N-acetylcysteine in an ex vivo model of COPD exacerbation

**DOI:** 10.1186/s12931-016-0500-y

**Published:** 2017-01-24

**Authors:** Mario Cazzola, Luigino Calzetta, Francesco Facciolo, Paola Rogliani, Maria Gabriella Matera

**Affiliations:** 1 Department of Systems Medicine, Chair of Respiratory Medicine, University of Rome Tor Vergata, Via Montpellier 1, 00133 Rome, Italy; 2 Regina Elena National Cancer Institute, Thoracic Surgery Unit, Rome, Italy; 3 Department of Experimental Medicine, Unit of Pharmacology, Second University of Naples, Naples, Italy

**Keywords:** Anti-inflammatory effect, Anti-oxidant effect, COPD, Lipopolysaccharide, N-acetylcysteine

## Abstract

**Background:**

Oxidative stress is recognized to be one of predisposing factor in the pathogenesis of COPD. The oxidant/antioxidant imbalance is significantly pronounced in patients with COPD exacerbation. N-acetylcysteine (NAC) seems to be able to reduce COPD exacerbations by modulating the oxidative stress in addition to its well-known mucolytic activity, but there are discordant findings on the actual anti-oxidant activity of NAC.

**Methods:**

The anti-oxidant effect of NAC and its impact on the inflammatory response have been pharmacologically characterized on a human ex vivo model of COPD exacerbation induced by lipopolysaccharide (LPS).

**Results:**

NAC prevented the desensitization induced by LPS incubation on the contractile tone in linear concentration-response manner. Concentrations of NAC ≥1 μM reduced the pro-oxidant response (peroxidase activity, hydrogen peroxide, malondialdehyde, nitric oxide), and improved the anti-oxidant response (total anti-oxidant capacity, glutathione, superoxide dismutase) induced by LPS. Lower concentrations of NAC (<1 μM) did not modulate the bronchial oxidative imbalance. Concentrations of NAC ≥300 μM inhibited the inflammatory response (release of IL-1β, IL-8, and TNF-α) of human airways induced by the overnight stimulation with LPS, whereas lower concentrations of NAC (≥1 μM) were sufficient to reduce the release of IL-6 elicited by LPS. Both the anti-oxidant effect and the anti-inflammatory effect of NAC were inversely correlated with the release of NKA.

**Conclusions:**

The findings of this study suggest that NAC may have a role in modulating the detrimental effect induced by LPS in course of COPD exacerbation. It may elicit both anti-oxidant and anti-inflammatory effects when administered at high concentrations.

## Background

Oxidative stress is recognized to be one of the critical factors in the pathway by which cigarette smoke exposure leads to disease [1]. It is not surprising, therefore, that there is solid evidence of increased oxidative stress in the airways of patients with chronic obstructive pulmonary disease (COPD), which is increased further in severe and very severe exacerbations of the disease and is associated with increased neutrophil influx and interleukin (IL)-8 levels during exacerbations [2]. The documentation that the glutathione (GSH) levels are increased in the bronchoalveolar lavage fluid (BALF) of patients with stable COPD and are reduced during exacerbations compared with stable COPD [2] suggests that the increase of lung GSH may be an attempt, albeit insufficient, to counter excess oxidant production, attempt totally inadequate during exacerbations due to the excessive production of reactive oxygen species (ROS). It is obvious that in this clinical condition, we must always try to increase the GSH levels [1].

Two pivotal trials and a recent meta-analysis have shown that N-acetylcysteine (NAC) is able to prevent COPD exacerbations [3–5]. NAC acts as a mucolytic, antioxidant and anti-inflammatory agent [6]. The antioxidant activity of NAC may be both direct (the free sulfhydryl group may serve as a ready source of reducing equivalents) and indirect (through replenishment of intracellular GSH levels) antioxidant effects [1]. However, there are several conflicting in vitro and in vivo findings on the real anti-oxidant activity of NAC, which, in any case, seems to be mainly dose-dependent [7].

Therefore, the aim of this study has been to pharmacologically characterize the anti-oxidant effect of NAC and its impact on the inflammatory response in a human ex vivo model of COPD exacerbation induced by lipopolysaccharide (LPS). Actually, since respiratory infections are associated with the majority of COPD exacerbations and their severity, especially those with bacterial co-infection, inhaled LPS provocation has been demonstrated to be an effective model of COPD exacerbation [8, 9]. LPS, also called endotoxin, is a major pro-inflammatory glycolipid component of the outer cell membrane of Gram-negative bacteria associated with the development and/or progression of many types of lung diseases including COPD [10]. LPS inhalation is related with elevation of neutrophils, macrophages and certain cytokines/chemokines in sputum and BALF, and similar inflammatory changes are observed during exacerbations COPD.

## Methods

### Tissue collection and preparation

Macroscopically normal airways were obtained from 6 moderate-to-severe COPD patients (4 male, 2 female, age 65.2 ± 5.8 years old) undergoing surgery for lung cancer. Samples were taken from an area as far as possible from the malignancy. Tissues were placed into Krebs-Henseleit buffer solution (KH) (NaCl, 119.0 mmol; KCl, 5.4 mmol; CaCl2, 2.5 mmol; KH2PO4 mmol, 1.2 mmol; MgSO4, 1.2 mmol; NaHCO3, 25.0 mmol; glucose, 11.7 mmol; pH 7.4) containing indomethacin (5 μM) and transported to the Laboratory of Respiratory Clinical Pharmacology at the University of Rome Tor Vergata (Italy) from a surrounding hospital.

Ethical approval (RS 60.15, 2015) and informed consent were consistent with the 2009 National Committee of Bioethics, National Committee of Bio-safety, Biotechnology and Sciences (Italy) recommendations on the collection of biological samples for research purposes, the 2010 Italian ethical and legal recommendations concerning the biobank and the research biorepository (Istituto Nazionale dei Tumori — Independent Ethics Committee, 2010), and the Comitato Nazionale per la Biosicurezza, le Biotecnologie e le Scienze per la Vita (Raccolta di campioni biologici a fini di ricerca, consenso informato, 2009; available at: http://www.governo.it/bioetica/gruppo_misto/Consenso_Informato_allegato_Petrini_2009.pdf).

In our laboratory, airways were cut into rings (thickness 1–2 mm; diameter 4–6 mm) and transferred into a 10 ml High Tech 8 Channels Manual Compact Organ Bath system (Panlab Harvard Apparatus, Spain) containing KH-buffer (37 °C) and aerated with O_2_/CO_2_ (95:5%). Tissues were allowed to equilibrate and the KH buffer was constantly changed.

### Preparation of drugs

The following compounds were used: GSH, indomethacin, LPS from *E. coli* 0111:B4 and NAC. Compounds were dissolved in distilled water except for indomethacin, which was dissolved in ethanol and then diluted in a KH buffer. The maximal amount of ethanol (0.02%) did not influence isolated tissue responses. Appropriate dilutions were obtained in freshly prepared medium and stock solutions stored at —80 °C until use. NAC dilutions were prepared daily before experiments.

### Tension measurement

Bronchial rings were connected to isometric force transducers Fort25 (WPI, UK). The signal was amplified by Bridge Amplifiers for Biopac system, recorded and analyzed with the Biopac interface software (16 + 16 channels). Tissues were mounted on hooks, and attached with thread to a stationary rod and the other tied with thread to an isometric force displacement transducer. Airways were allowed to equilibrate for 90 min flushing with fresh KH buffer solution every 10 min. Passive tension was determined by gentle stretching of tissue (0.5–1.0 g) during equilibration. The isometric change in tension was measured by the transducer and the tissue responsiveness assessed by electrical field stimulation (EFS) at 25 Hz. After that, rings ware washed three times and allowed to stabilize.

### COPD exacerbation model

Bronchial tissues were incubated overnight with KH buffer solution (negative control) or LPS (100 ng/ml, positive control) in order to mimic ex vivo the condition of airways during COPD exacerbation in vivo [11–13].

Some LPS-incubated tissues were pre-treated with increasing concentrations of NAC from very low to high concentrations (10 nM, 100 nM, 1 μM, 3 μM, 10 μM, 30 μM, 100 μM, 300 μM, 1 mM and 10 mM) in order to reproduce in the bath the plasmatic bioavailability following low and high oral doses of NAC [14–16]. Further tissues were pre-treated with reduced GSH (100 μM), as control for anti-oxidant activity [17–19].

The day after, bronchial rings were mounted into the isolated organ bath system and connected to the isometric force transducers for recording the contractile response of airway smooth muscle (ASM) in response to transmural stimulation [20, 21].

### Transmural stimulation

Transmural EFS was performed by placing tissues between two wire platinum electrodes (20 mm apart, Panlab Harvard Apparatus, Spain), connected to a 3165 multiplexing pulse booster stimulator (Ugo Basile, VA - Italy). Reference standard contraction was then assessed for every bronchial ring by stimulating samples with a train of 25Hz EFS impulses (10 V, 10 s, 0.5 ms). After that, bronchial rings were contracted by EFS at increasing frequencies (1, 3, 10, 25 and 50 Hz) in order to simulate the vagal firing (parasympathetic pathway) normally observed in human in vivo at physiological frequency range [12, 22].

### Pro- anti-oxidant factors and cytokines quantification

The supernatant from all treatments was collected in order to assess the influence of NAC on the pro- and anti-oxidant response and on the release of cytokines.

The pro-oxidant response was assessed by quantifying the activity of peroxidase and the concentrations of hydrogen peroxide, malondialdehyde and nitric oxide. The protective response to oxidative stress was investigated by measuring the total anti-oxidant capacity, GSH and the superoxide dismutase activity. The levels of cytokines such as IL-1β, IL-6, IL-8 and tumor necrosis factor (TNF)-α were also determined and compared with the respiratory burst induced by the incubation with LPS [7, 23, 24].

The quantification of pro- and anti-oxidant factors and of cytokines were carried out by performing colorimetric/fluorometric and ELISA assays characterized by high sensitive detection limits and high specificity, in agreement with manufacturers’ datasheets (BioCat, Heidelberg, Germany).

### Neurokinin A quantification

The collected supernatant was also used to quantify the levels of neurokinin A (NKA) in response to LPS incubation and NAC treatment. The quantification of NKA was performed via ELISA assays in agreement with manufacturers’ datasheets (BioCat, Heidelberg, Germany).

### Data analysis

The contractile responses will be expressed as a percentage of the maximal effect (E_max_) induced by EFS in control bronchi. Appropriate curve fitting to a sigmoidal model was used to calculate the effect (E), the E_max_, the dose inducing n% E_max_ (EC_n_). The equation used was: log (agonist; antagonist) vss response, Variable slope, expressed as Y = Bottom + (Top - Bottom)/{1 + 10^[(LogEC_50_ - X)*HillSlop2]}. For statistical analysis of the potency, the pEC_50_ value was used where pEC_50_ = −logEC_50_ [25].

Linear regression was used for analyzing the effectiveness of NAC as described elsewhere. The levels of NKA were correlated (Pearson’s correlation and 95% CI) with the pro- anti-oxidant factors and cytokines released by isolated bronchi [25].

Experiments were performed on *n* = 4 bronchi collected from different subjects and values presented as mean ± SEM. In agreement with tissues availability and vitality, each single treatment was carried out by using specimens collected from the same patient, and experiments were repeated 4 times in samples originating from 4 different donors. When the amount of sample from one subject did not permit to perform all the treatments, including the pre-treatment with increasing concentrations of NAC, the remaining treatments were carried out in specimens collected from other patients in parallel with further positive and negative controls.

The evaluation of the pro- and anti-oxidant activity and the quantification of cytokines were normalized for 100 mg of bronchial tissue and carried out by performing experiments repeated in triplicate.

The statistical significance was assessed by the t-test or the two-way analysis of variance (ANOVA), and the level of statistical significance will be defined as *P* < 0.05. Data analysis was performed by using Prism 5 software (GraphPad Software Inc, CA, USA).

## Results

### Influence of NAC on the bronchial desensitization induced by LPS

**Fig. 1:**
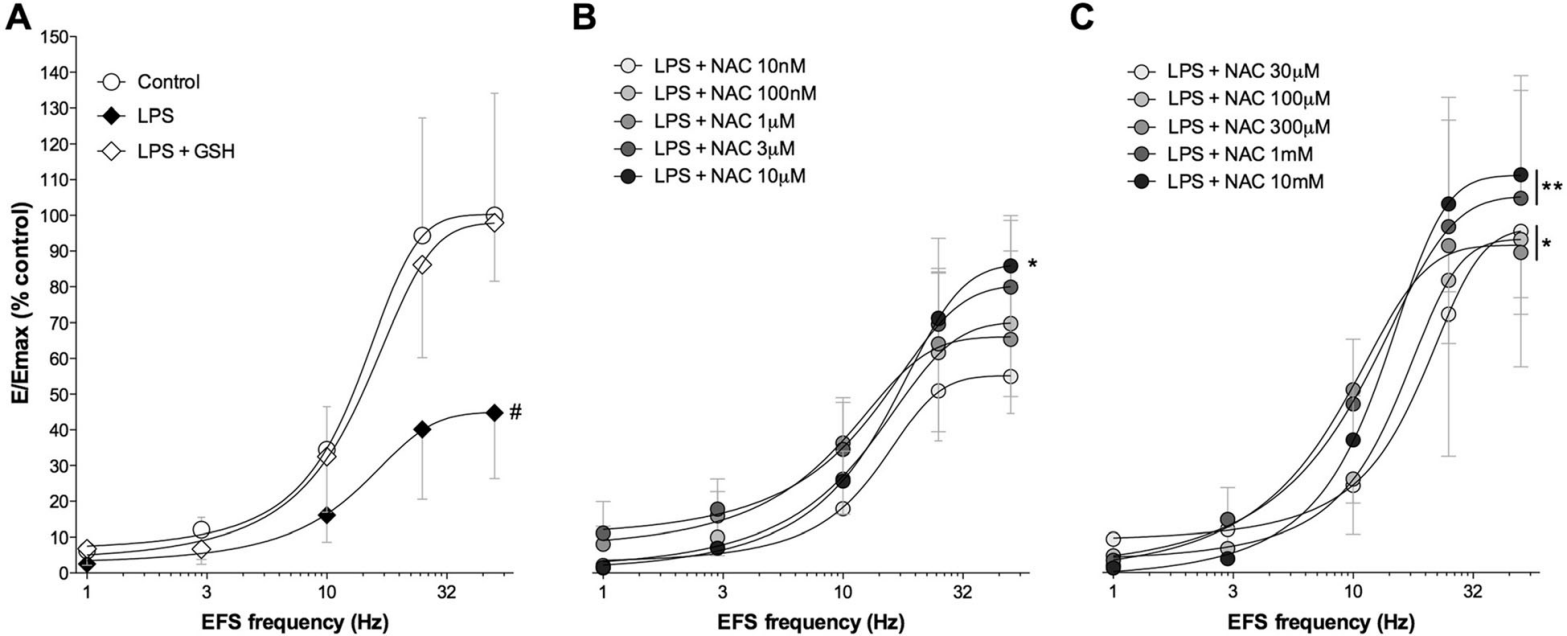
Frequency-response curves produced by EFS ranging from 1 Hz to 50 Hz in control bronchi and after overnight incubation with LPS (100 ng/ml), alone or together with GSH (100 μM) (**a**). Influence of increasing concentrations of NAC (**b** 10 nM to 10 μM; **c** 30 μM to 10 mM) on the desensitizing effect induced by LPS (100 ng/ml). Data are expressed as the mean ± SEM of *n* = 4 segmental bronchi from different subjects. # *P* < 0.05 vs. control bronchi: * *P* < 0.05 and **P < 0.01 vs. LPS-incubated bronchi (statistical significance assessed by two-way ANOVA for comparison among control, LPS, LPS + GSH and LPS + NAC treatments)

The overnight incubation with LPS (100 ng/ml) induced a significant (*P* < 0.05 vs. control) desensitization of human isolated bronchial rings on the contractile response induced by EFS, whereas GSH (100 μM) completely abolished this effect (*P* > 0.05 vs. control) (E_max_: control 100.20 ± 21.32%, LPS 44.92 ± 12.70%, LPS + GSH 97.90 ± 7.75%; Fig. 1a).

The concomitant incubation with NAC administered at concentrations ranging from 10 nM to 3 μM did not modulate the desensitizing effect induced by LPS (*P* > 0.05 vs. LPS-incubated bronchi). On the other hand, higher concentrations of NAC significantly prevented the LPS-induced desensitization (*P* < 0.05 vs. LPS-incubated bronchi), thus restoring the physiological contractile response elicited by EFS in isolated airways (NAC 10 μM to 300 μM: E_max_ 86.39 ± 9.92% to 96.16 ± 27.43%; NAC 1 mM to 10 mM: E_max_ 105.20 ± 21.42% to 111.10 ± 18.55%; Fig. 1c and b).

**Fig. 2:**
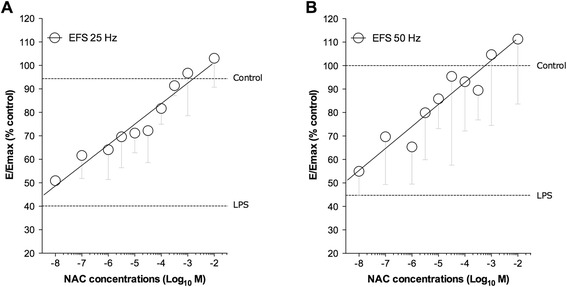
Linear concentration-response curves by NAC on the desensitization induced by overnight incubation of human isolated bronchi with LPS (100 ng/ml). Data are expressed as the mean ± SEM contractile response to EFS delivered at 25 Hz (**a**) and 50 Hz (**b**) in *n* = 4 segmental bronchi from different subjects

The analysis of potency was carried out for EFS inducing ≈ E_max_ in control airways, specifically EFS 25 Hz and 50 Hz. NAC prevented the desensitization induced by LPS incubation in a linear concentration-response manner, with analogous pEC_50_ values for both EFS 25 Hz and 50 Hz (5.45 ± 0.24 and 5.77 ± 0.42, respectively; *P* > 0.05) (Fig. 2).

### Influence of NAC on the pro- and anti-oxidant response in LPS-incubated airways

**Fig. 3:**
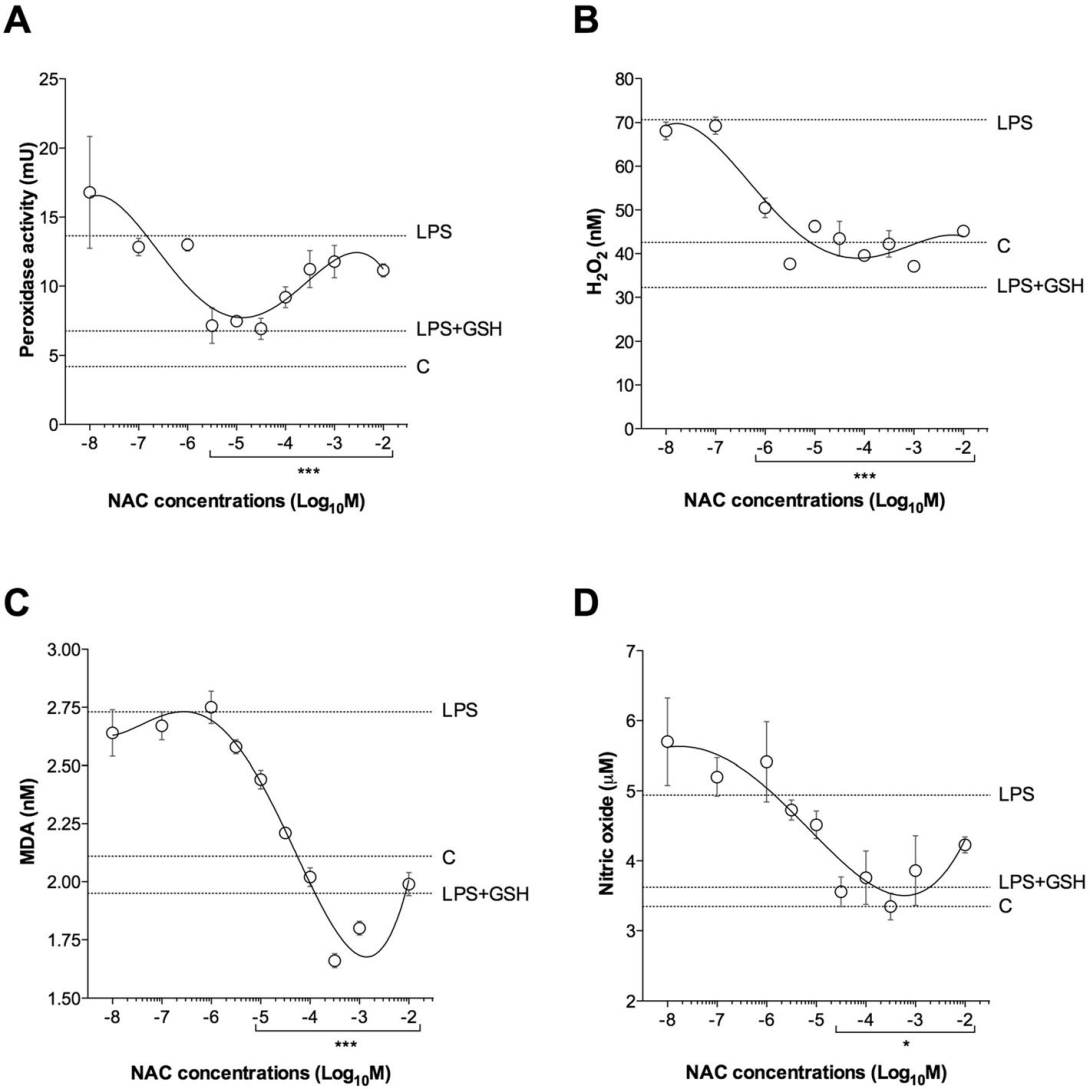
Influence of NAC on the pro-oxidant response (**a** peroxidase activity, **b** H_2_O_2_ concentrations, **c** malondialdehyde [MDA] concentrations and **d** nitric oxide concentrations) induced by overnight incubation of human isolated bronchi with LPS (100 ng/ml). Horizontal dotted lines indicate the pro-oxidant response in control bronchi (**c**), in bronchi treated with LPS alone or in the presence of GSH. Data are expressed as the mean ± SEM of experiments repeated in triplicate. * *P* < 0.05 and ***P < 0.001 vs. LPS-incubated bronchi (statistical significance assessed by two-way ANOVA)

The overnight incubation with LPS (100 ng/ml) significantly increased the pro-oxidant response of human isolated bronchi (peroxidase activity: +101.40 ± 3.58%; hydrogen peroxide: +66.03 ± 2.79%; malondialdehyde +29.36 ± 0.74%; nitric oxide: +46.59 ± 10.01; *P* < 0.001 vs. control), and this effect was reduced by GSH (100 μM; *P* < 0.001 vs. LPS-incubated bronchi). NAC administered at medium-to-high concentrations (from 1 μM to 10 mM) significantly reduced the pro-oxidant response induced by LPS (peroxidase activity: −32.03 ± 2.95%; hydrogen peroxide: −39.50 ± 1.56%; malondialdehyde −29.0.5 ± 0.54%; nitric oxide: −24.06 ± 1.79%; *P* < 0.05 vs. LPS-incubated bronchi) (Fig. 3).

**Fig. 4:**
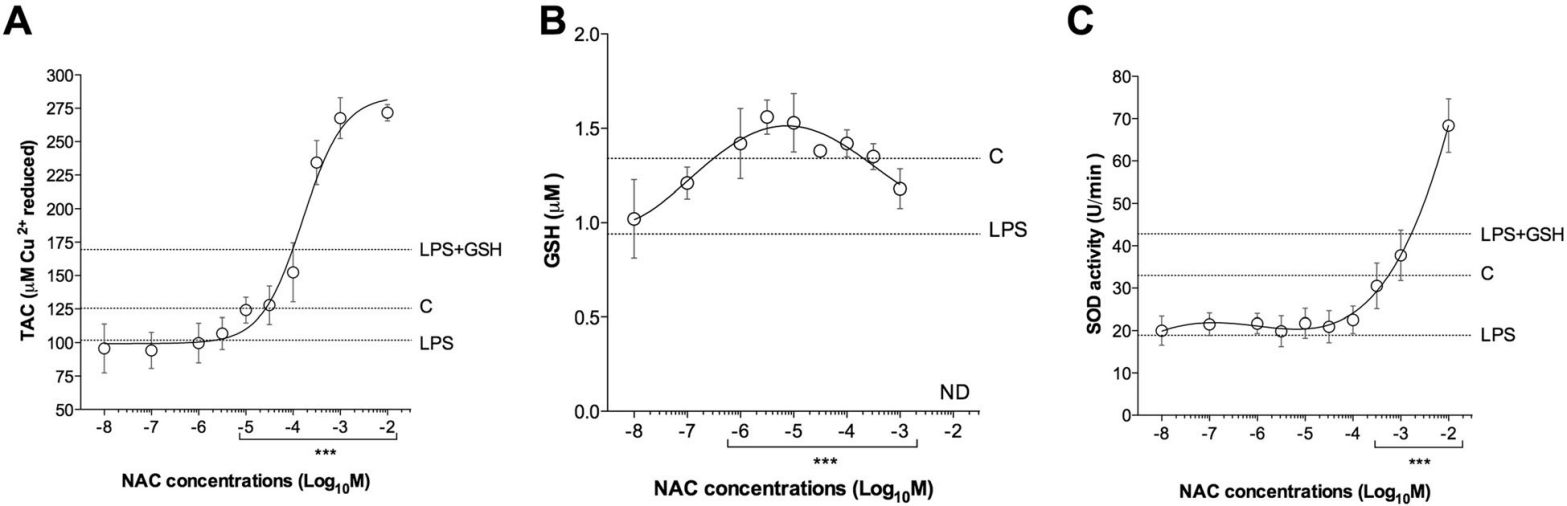
Influence of NAC on the anti-oxidant response (**a** total anti-oxidant capacity [TAC], **b** GSH concentrations and **c** superoxide dismutase [SOD] activity) induced by overnight incubation of human isolated bronchi with LPS (100 ng/ml). Horizontal dotted lines indicate the anti-oxidant response in control bronchi (**c**), in bronchi treated with LPS alone or in the presence of GSH. In B the LPS + GSH treatment has been not carried out in order to not influence the GSH quantification. Data are expressed as the mean ± SEM of experiments repeated in triplicate. ***P < 0.001 vs. LPS-incubated bronchi (statistical significance assessed by two-way ANOVA). ND: not detectable

The incubation with LPS (100 ng/ml) significantly reduced the anti-oxidant response of human isolated bronchi (total anti-oxidant capacity: −18.85 ± 1.94%; GSH: −29.76 ± 2.87%; superoxide dismutase: −44.82 ± 6.15%). The pre-treatment with GSH (100 μM) significantly prevented the anti-oxidant impairment response (*P* < 0.001 vs. LPS-incubated bronchi). NAC administered at medium-to-high concentrations (from 1 μM to 10 mM) was effective in preventing the reduced anti-oxidant response induced by LPS (total anti-oxidant capacity: +92.91 ± 6.61%; GSH: +49.56 ± 3.53%; superoxide dismutase: +150.40 ± 19.51%; *P* < 0.001 vs. LPS-incubated bronchi) (Fig. 4).

### Influence of NAC on the release of cytokines in LPS-incubated airways

**Fig. 5:**
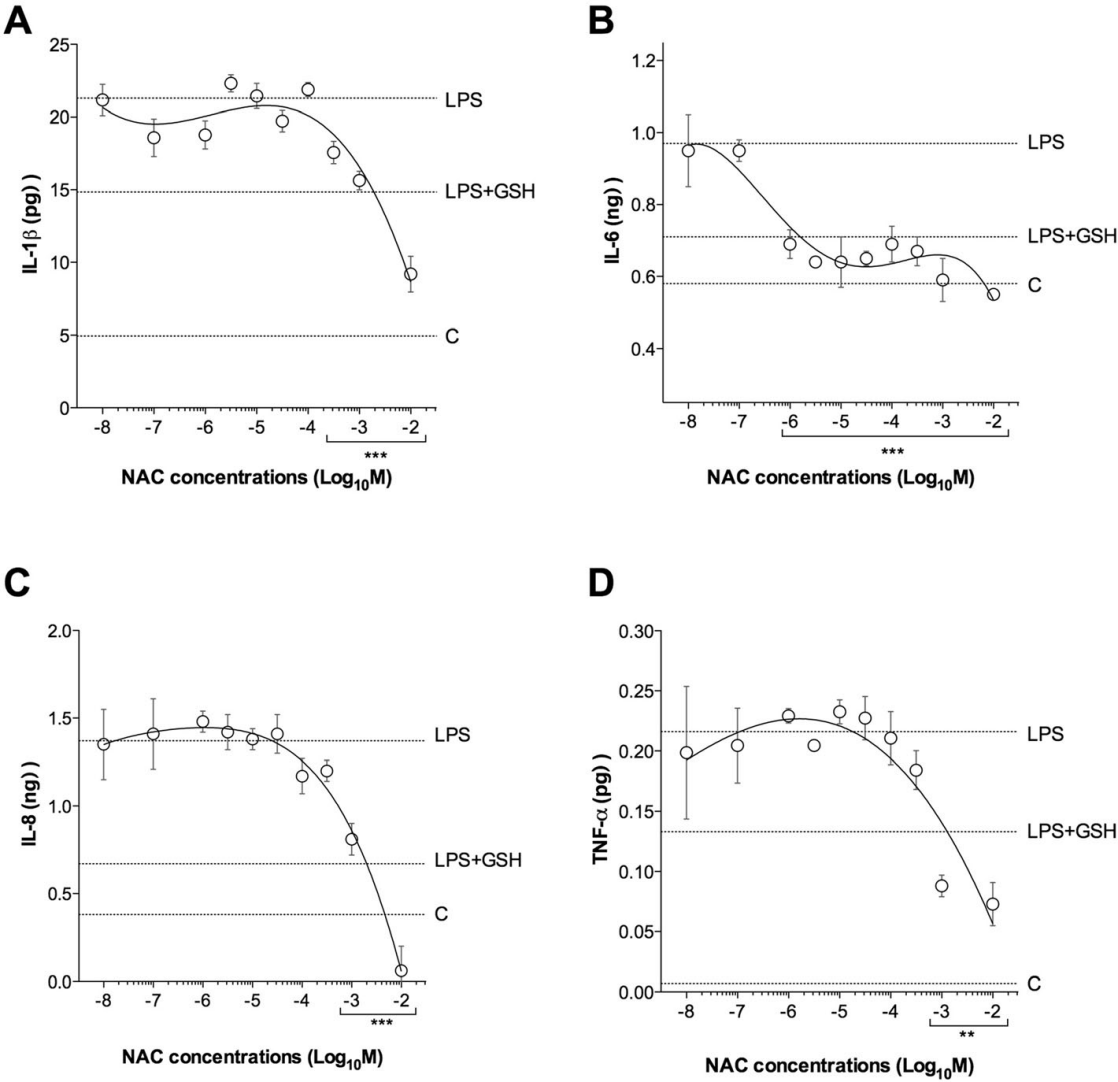
Influence of NAC on the cytokines release (**a** IL-1β concentrations, **b** IL-6 concentrations, **c** IL-8 concentrations and **d** TNF-α concentrations) induced by overnight incubation of human isolated bronchi with LPS (100 ng/ml). Horizontal dotted lines indicate the cytokines concentrations in control bronchi (**c**), in bronchi treated with LPS alone or in the presence of GSH. Data are expressed as the mean ± SEM of experiments repeated in triplicate. **P < 0.01 and ***P < 0.001 vs. LPS-incubated bronchi (statistical significance assessed by two-way ANOVA)

The overnight incubation with LPS (100 ng/ml) significantly increased the release of cytokines in human isolated airways (IL-1β: +331.24 ± 17.03%; IL-6: +67.32 ± 7.28%; IL-8: +260.00 ± 17.50%; TNF-α: +3166.26 ± 545.77%; *P* < 0.001 vs. control), and this effect was reduced by GSH (100 μM; *P* < 0.001 vs. LPS-incubated bronchi). NAC administered at high concentrations (from 300 μM to 10 mM) significantly inhibited the release of IL-1β (−33.62 ± 2.10%), IL-8 (−68.11 ± 17.34%) and TNF-α (−62.82 ± 10.34%), when compared with LPS-incubated bronchi (*P* < 0.01). Lower concentrations of NAC (from 1 μM to 10 mM) were significantly (*P* < 0.001) effective in reducing the release of IL-6 (−33.16 ± 2.02%) induced by the stimulation with LPS (Fig. 5).

### Influence of NAC on the release of neurokinin A in LPS-incubated airways

**Fig. 6:**
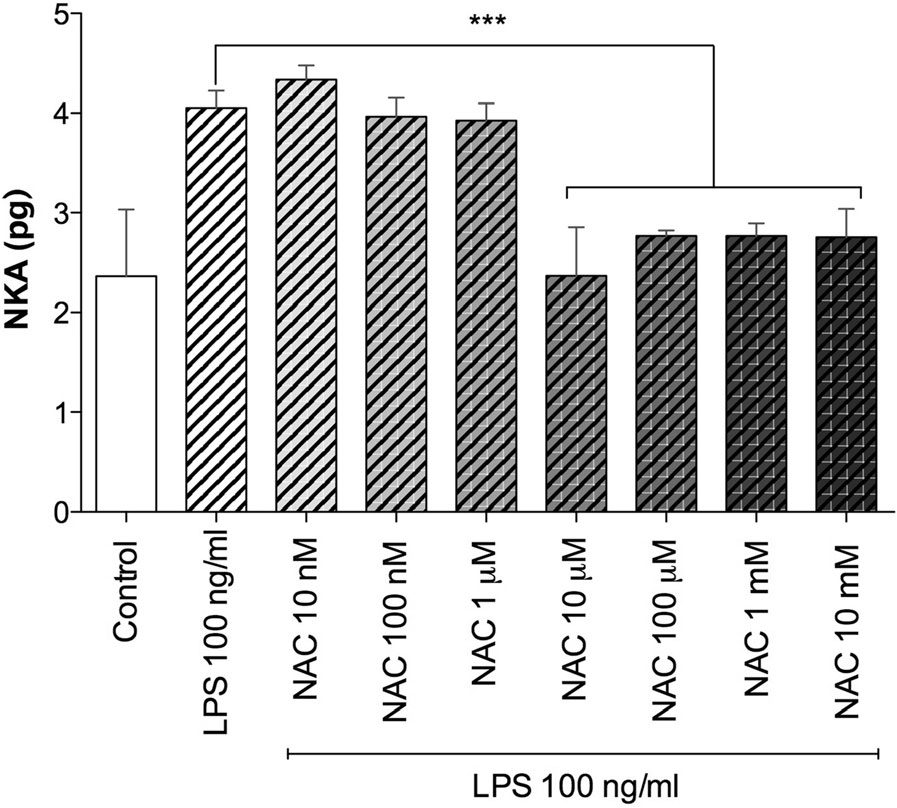
Influence of NAC on the release of neurokinin A (NKA) induced by overnight incubation of human isolated bronchi with LPS. Data are expressed as the mean ± SEM of experiments repeated in triplicate. ***P < 0.001 vs. LPS-incubated bronchi (statistical significance assessed by two-way ANOVA)

The overnight incubation with LPS (100 ng/ml) induced a significantly release of NKA (+71.75 ± 3.04%; *P* < 0.001 vs. control). NAC administered at high concentrations (from 10 μM to 10 mM) significantly inhibited the release of NKA (−31.90 ± 2.37%), when compared with LPS-incubated bronchi (*P* < 0.001) (Fig. 6).

The release of NKA was positively correlated with the levels of pro-oxidant factors (peroxidase activity, H_2_O_2_, malondialdehyde [MDA], nitric oxide [NO]; *P* < 0.05) and concentrations of the cytokine IL-6 (*P* < 0.05), whereas it was negatively correlated with the levels of the anti-oxidant factor GSH (*P* < 0.05). No significant correlation was found for total anti-oxidant (TAC), superoxide dismutase (SOD), IL-1β, IL-8, and TNF-α (*P* > 0.05) (Table 1).

**Table 1:** Correlation between NKA concentrations and levels of pro-oxidant factors, anti-oxidant factors and cytokines after overnight incubation with LPS (100 ng/ml)

NKA vs:	Pearson r	95% CI	*P*
Peroxidase activity	0.85	0.47 - 0.96	***
H_2_O_2_	0.84	0.44 - 0.96	**
MDA	0.63	0.01 - 0.90	*
NO	0.83	0.41 - 0.96	**
TAC	−0.49	−0.86 - 0.20	ns
GSH	−0.63	−0.91 - 0.058	*
SOD	−0.28	−0.77 - 0.43	ns
IL-1β	0.13	−0.55 - 0.70	ns
IL-6	0.82	0.4 0–0.96	**
IL-8	0.30	−0.40 - 0.78	ns
TNF-α	0.19	−0.50 - 0.73	ns

* *P*<0.05, ** *P*<0.01, *** *P*<0.001, ns: not significant. *GSH* glutathione, *IL* interleukin, *MDA* malondialdehyde,* NKA* neurokinin A, *NO* nitric oxide, *ROS* reactive oxygen species, *SOD* superoxide dismutase, *TAC* total anti-oxidant capacity, *TNF-α* tumor necrosis factor-α

## Discussion

The role of ASM has been the subject of provocative commentaries, proposing that ASM is a useless, vestige of phylogeny, perhaps retained only because of its common ontogenetic origin with the gut [26–29]. Nevertheless, an elegant analysis showed how ASM is useful [26]. In fact, ASM is part of an alarm system, akin to allergic pruritus, warning us of exposures to dangerous toxins and allergens to which we might otherwise be sensitive. The primary function of ASM is to contract, which regulates airway tone and bronchial narrowing [27, 30–32]. Although complete airway closure is fatal, uncomfortable partial closure is likely to provide life-preserving function [26]. Further roles for ASM consist of peristalsis to assist exhalation and mucus propulsion, promotion of lymphatic and venous flow, ventilation/perfusion matching, protection of peripheral lung and airway architecture, enhancement of the effectiveness of cough, optimization of anatomic dead space volume, and modulation of immune and proliferative responses [27, 30]. Therefore, while no single role for ASM contributes a large adaptive advantage, cumulatively they provide a distinct benefits [26]. Taken together, these evidences clearly indicate that a physiological bronchial contractility is necessary to protect the airways from the insult of external pathogens.

The results of our study demonstrate that the overnight challenge of human isolated airways with LPS 100 ng/ml induces bronchial desensitization, and NAC counteracts this desensitization by restoring the physiological contractility of ASM, at least in an ex vivo model of COPD exacerbation. This finding suggests that NAC may preserve the normal bronchoreactivity in COPD patients that are chronically colonized by Gram-negative bacteria, leading to protection against exacerbations.

Previous researches reported that the acute challenge of human isolated tissues with LPS administered at 300 ng/ml for 2 h induced bronchial hyperresponsiveness (BHR) [12, 33]. Actually, the acute activation of sensory c-fibers by endotoxin and capsaicin provokes BHR due to the facilitation of cholinergic pathway driven by vagus nerve [33, 34]. Thus, although the desensitizing effect of overnight exposure to LPS may appear paradoxical, it had to be expected since also multiple challenges with capsaicin led to bronchial desensitization [21].

Results of our experiments suggest that also other pharmacological characteristics of NAC, such as the anti-oxidant and anti-inflammatory activities, may explain the protective role with regard of COPD exacerbations.

GSH is a carrier of active thiol group in form of a cysteine (Cys) residue, and acts as an antioxidant by interacting with reactive oxygen/nitrogen species and electrophiles and operating as a cofactor for various enzymes [1]. The loss of antioxidant capacity in oxidatively stressed cells/tissues is related to a decrease in GSH and/or its precursor Cys. Cys cannot be used as a GSH precursor due to its toxicity at high concentrations, and also because it is susceptible to metabolism and rapid oxidation, generating the inactive disulphide cystine (Cys–Cys) [35, 36]. GSH concentrations are increased in BALF of patients with stable COPD, and reduced in course of exacerbations [2]. This evidence suggests that the increase of lung GSH may be an attempt to counteract the excessive oxidant production, attempt totally inadequate during exacerbations due to the excessive production of ROS [1].

NAC acts as a precursor for the substrate Cys in the synthesis of GSH, by delivering sulfhydryl moieties for utilization in biological processes [37]. In fact, NAC may restore the pool of intracellular GSH that has been depleted following increased status of oxidative stress and inflammation in course of COPD exacerbation, by modulating the activity of redox-sensitive cell-signaling and transcription pathways such as the Nuclear Factor-kB [38].

Although from a strict biochemical point of view the antioxidant activity of NAC has been proved to be both direct (the free sulfhydryl group may serve as a ready source of reducing equivalents) and indirect (through replenishment of intracellular GSH levels), the real impact of NAC on the imbalance between oxidative stressors and anti-oxidants in the human bronchial tissue has been never investigated before. Here, we have ultimately demonstrated that NAC is effective in improving the antioxidant activity and reducing ROS elevation in human airways in course of a validated model of COPD exacerbation.

The possible pathways through which NAC can modulate inflammation have been suggested taking into account the data obtained in in vitro studies, but to date discordant findings still exist in vivo [7]. Sadowska and colleagues [7] suggested that this discrepancy might be explained by the fact that the effects of NAC are cell specific and concentration-dependent. The authors also highlighted that the concentrations necessary to elicit anti-inflammatory effects are higher than those required for an anti-oxidant response, and that higher doses are needed to achieve acute anti-oxidant and anti-inflammatory effects [7]. Our results confirm these hypotheses. In fact, we have demonstrated that the concentrations of NAC necessary to elicit anti-inflammatory effects in human bronchi stimulated by LPS were at least one logarithm higher than those required to improve the antioxidant capacity. This gradient of effectiveness indicates that perhaps the anti-inflammatory effect of NAC is secondary to its antioxidant activity.

Nevertheless, we cannot omit to highlight that NAC administered at only 1 μM, a concentration able to induce effective anti-oxidant effect, was also enough to prevent IL-6 elevation. This result implies that, while higher concentrations of NAC may modulate the release of cytokines such as IL-1β, IL‐8 and TNF‐α by inhibiting oxidative stress, some further mechanisms that go beyond the anti-oxidant activity may regulate the IL-6 increase, in particular when NAC is administered at lower concentrations.

Specifically, our findings suggest that NAC might modulate the neurogenic inflammatory response induced by LPS through the inhibition of NKA release, and thus reduce IL-6 increase. Although the role of LPS in increasing the neurogenic inflammation has been widely demonstrated in airways [12, 33, 34], we cannot exclude that IL-6 itself might induce a further enhancement of NKA release, as demonstrated in the hypothalamic-pituitary axis [39]. Intriguingly, our study confirms that very low concentrations of NKA might play a pivotal role in fine-tuning BHR in human tissues.

Our study cannot discriminate whether the neurogenic inflammation induced by LPS led to oxidative stress or vice versa. Nevertheless, it is clear that both ROS formation and neurogenic inflammation are critical consequences of an exposure to LPS, a typical condition of acute COPD exacerbation that is also characterized by significantly increased levels of IL-6 [40].

Overall, the findings of the present study corroborate the opinion that NAC may be effective in reducing COPD exacerbations not only via its indirect anti-oxidant activity, but also by inducing an anti-inflammatory effect secondary to an improved anti-oxidant capacity. Furthermore, they support the possibility that NAC can even act by modulating the neurogenic inflammation, a deleterious condition that may support the vicious circle between oxidative stress and inflammation.

Plasma concentrations of NAC reaches values of ≈ 5 μM after oral administration of 200 mg, ≈16 μM after oral administration of 600 mg, and ≈ 35 μM after an oral administration of 1,200 mg [14, 41, 42]. Therefore, the results of our study confirm that NAC administered at 600 mg may elicit prevalently anti-oxidant activity, whereas higher doses are necessary to produce anti-inflammatory effect. This consideration is in agreement with the results of a recent meta-analysis advising that in a patient suffering from COPD with an objective confirmation of airways obstruction NAC should always be administered at a dose ≥1200 mg per day to prevent COPD exacerbations, while in a patient with chronic bronchitis, but without airway obstruction, a regular treatment of 600 mg per day seems to be sufficient [5].

## Conclusion

The findings of this study suggest that NAC may have a role in modulating the detrimental effect induced by LPS in course of COPD exacerbation. It may elicit both anti-oxidant and anti-inflammatory effects when administered at high concentrations.

## Abbreviations

ASM: Airway smooth muscle
BALF: Bronchoalveolar lavage fluid
BHR: Bronchial hyperresponsiveness
COPD: Chronic obstructive pulmonary disease
Cys: Cysteine
EFS: Electrical field stimulation
GSH: Glutathione
IL: Interleukin
LPS: Lipopolysaccharide
MDA: Malondialdehyde
NAC: N-acetylcysteine
NKA: Neurokinin A
NO: Nitric oxide
ROS: Reactive oxygen species
SOD: Superoxide dismutase
TAC: Total anti-oxidant capacity
TNF-α: Tumor necrosis factor-α
